# Loneliness in Personality Disorders

**DOI:** 10.1007/s11920-022-01368-7

**Published:** 2022-10-01

**Authors:** Matthias A. Reinhard, Tabea Nenov-Matt, Frank Padberg

**Affiliations:** Department of Psychiatry and Psychotherapy, LMU University Hospital Munich, Nussbaumstr. 7, 80336 Munich, Germany

**Keywords:** Loneliness, Perceived social isolation, Social network, Personality disorders, Alternative model of personality disorders, Psychotherapy

## Abstract

***Purpose of Review*:**

Loneliness is a common experience in patients with personality disorders (PDs) that are characterized by impairment in self (identity, self-direction) and interpersonal functioning (empathy, intimacy). Here, we review studies assessing the association of loneliness with PD or PD traits including DSM-5’s Alternative Model of PD (AMPD).

***Recent Findings*:**

The number of loneliness studies varied greatly among different PDs with most studies conducted in borderline PD. Across PDs, loneliness was associated with the severity of psychopathological symptoms and with several AMPD trait domains. Consequently, loneliness may contribute to PD severity and further impair personality functioning.

***Summary*:**

Loneliness and PD share intra- and interpersonal factors (i.e., increased rejection sensitivity, information processing biases, social withdrawal) and common origins in childhood maltreatment that may explain their close association. Future research needs to investigate mechanisms on how loneliness and core characteristics of PD mutually reinforce each other in order to therapeutically address loneliness in PD.

## Introduction

Loneliness is an aversive feeling of being alone that arises from the subjective perception of unsatisfactory social relationships [1]. Most people experience loneliness at some point during their life [2] with particularly vulnerable phases in young adulthood and old age [3]. In a recent Europe-wide study, 9% of respondents said they feel occasionally lonely [4]. Loneliness shows great stability across the life span comparable to personality traits [5]. Furthermore, loneliness has a negative impact on both physical and mental health [6–8]. In particular, patients with personality disorders (PDs) commonly report feeling lonely and disconnected [9••]. Interestingly, compared to other mental health diagnostic groups, a mixed sample of PDs was even found to be most affected by loneliness [10].

PDs are characterized by enduring patterns of inner experiencing and behaving that markedly differ from what the individual’s culture expects and lead to impairment in self and interpersonal functioning [11]. PD patterns are inflexible, stable, distressing, and manifest in cognition, affectivity, interpersonal behavior, and/or impulse control [11]. As a consequence, patients with PD struggle in establishing and maintaining functional social relationships. Besides a categorical approach with ten specific PDs, DSM-5 has introduced a dimensional approach [11]: The Alternative Model for Personality Disorders (AMPD) defines impairment in self (identity, self-direction) and interpersonal functioning (empathy, intimacy) as core characteristics of PD (criterion A: personality functioning) that are further specified by dysfunctional personality trait domains (criterion B: negative affectivity, detachment, antagonism, disinhibition, psychoticism).

Patients with PD are at great risk to experience loneliness as suggested by a recent qualitative review that found that individuals with PD or PD traits experience an intense social disconnection from others [9••]. Here, we aimed at extending these findings by reviewing available empirical studies that assess loneliness in PD to guide further clinical research.

## Methods

Within a comprehensive literature search, original studies listed in PubMed until June 2022 were identified with the search terms “loneliness” AND “personality disorder,” “paranoid,” “schizoid,” “schizotypal,” “antisocial,” “borderline,” “histrionic,” “narcissistic,” “avoidant,” “dependent,” “obsessive–compulsive,” and “alternative model of personality disorders.” We considered studies that assessed categorical PDs and studies that assessed dimensional PD traits for this review. Only studies in English were considered with a focus on research published within the past 5 years. Furthermore, the reference lists of studies were screened for further studies of interest. In total, *N* = 35 studies were identified that assessed loneliness and PD or PD traits (paranoid: *n* = 5; schizoid: *n* = 1; schizotypal: *n* = 6; antisocial: *n* = 2; borderline: *n* = 7; histrionic: *n* = 1; narcissistic: *n* = 6; avoidant: *n* = 2; dependent: *n* = 3; obsessive–compulsive: *n* = 0; AMPD: *n* = 2).

## Measures of Loneliness

Though loneliness is common, its assessment in empirical research is hampered by the lack of objective measures and the use of self-report questionnaires. The most common measure in the studies reported here was the UCLA Loneliness Scale [12] already released in 1978 with its original 20 items and three subscales (i.e., emotional isolation, social isolation, feelings of loneliness) as well as its short forms. Over time, items asking directly about loneliness were rather excluded from subsequent versions of the UCLA Loneliness Scale to guard against biases of socially desirable responding. Other measures were the Loneliness Scale with 3 items [13] or the de Jong-Gierveld Loneliness Scale [14]. Recently, loneliness has been assessed with Ecological Momentary Assessment (EMA; e.g., “How lonely do you feel because of COVID-19 social distancing right now?” [15]).

## Paranoid Personality

Paranoid PD is characterized by a pervasive pattern of distrust and suspicion of others. Patients are reluctant to confide in others, resulting in withdrawal and supposedly increased risk of loneliness. Additionally, it can be assumed that the paranoid’s distrust and suspicion may impede overcoming loneliness as they hinder new and corrective experiences.

There is a lack of studies assessing loneliness in paranoid PD, yet studies with dimensional assessments of subclinical paranoia suggest an association between loneliness and paranoid traits. For instance, McIntyre et al. [16] found that loneliness was positively correlated with paranoid beliefs (*N* = 1135 university students). The result was replicated by Alsuhibani et al. [17] in students (*N* = 496) and a more representative population-based sample (*N* = 1519). Similarly, Bortolon et al. [18] found that loneliness contributed to paranoid thoughts in the general population (*N* = 728). Interestingly, Lamster et al. [19] showed that an experimentally induced reduction of loneliness via a false-feedback paradigm was associated with a reduction of paranoid thoughts in a non-clinical sample (*N* = 60). Vice versa, Gollwitzer et al. [20] found that recollecting an experience of loneliness increased paranoid thoughts (*N* = 222 non-clinical participants). Targeting loneliness and negative affect with cognitive reappraisal weakened the effect on paranoid thoughts (*N* = 196 non-clinical participants).

## Schizoid Personality

Schizoid PD describes a pervasive pattern of social detachment and withdrawal, restricted affectivity, and anhedonia. The resulting social isolation may appear like a willful choice. However, it can be assumed that minimal needs for belonging and occasional social support may occur that are not fulfilled and lead the schizoid patient to feel lonely. Due to the perceived impossibility of patients with schizoid PD to let someone close, it may be specifically difficult to overcome loneliness. Moreover, the distancing interpersonal impact may even reject others.

Empirical loneliness data for schizoid PD is scarce. Using the number of fulfilled schizoid PD diagnostic criteria as a dimensional score, Levi-Belz et al. [21] found that schizoid tendency was positively correlated with loneliness. Furthermore, both loneliness and schizoid tendency were associated with the lethality of suicide attempts in their sample of *N* = 336 psychiatric patients and controls.

## Schizotypal Personality

Schizotypal PD is characterized by a pervasive pattern of unusual beliefs and aberrant perceptual experiences (positive schizotypy), anhedonia and avolition (negative schizotypy), and disorganized thoughts/behavior. On the interpersonal level, patients with schizotypal PD report a lack of close friends and discomfort with close relationships outside their immediate family. Therefore, schizotypal PD may predispose to feel lonely due to social discomfort, social anxiety, and the experience of not being understood for their unusual beliefs.

Loneliness research in schizotypal personality mainly focuses on dimensional assessments of schizotypy. Benson and Park [22] found that loneliness significantly correlated with schizotypal personality traits, including the negative-interpersonal (i.e., no close friends, constricted affect), cognitive-perceptual, and disorganized facets (*N* = 62 college students). The strongest correlation was found for the negative-interpersonal facet. Similarly, Masucci et al. [15] observed that state loneliness due to COVID-19 restrictions, which was measured by EMA, was positively associated with the cognitive-perceptual schizotypal facet in undergraduates (*N* = 85) yet with inconsistent results for negative facets such as no close friends and constricted affect. Furthermore, loneliness seemed to contribute to psychotic-like symptoms such as hallucination- and delusion-like experiences during the COVID-19-pandemic (*N* = 850 participants [23]). Accordingly, Le et al. [24] found that loneliness interacted with cognitive-perceptual and disorganized schizotypy to predict psychotic-like symptoms in three samples with undergraduates (*N* = 78; *N* = 118) and male inpatients with substance use disorder (*N* = 48). In a recent study, Chau et al. [25] analyzed the association of loneliness with the diagnostic schizotypal traits in *N* = 2089 participants. They found that loneliness was strongly associated with the criteria “suspiciousness” and “no close friends” besides “odd beliefs or magical thinking,” “constricted affect,” and “excessive social anxiety.” However, besides their close association, Badcock et al. [26] emphasized that schizotypy and loneliness are still distinct constructs in a sample of students (*N* = 551).

## Antisocial Personality

Antisocial PD describes a pervasive pattern of disregard for and violation of the rights of others. Besides antisocial behavior, patients lack remorse about others’ distress and show a lack of empathy leading to difficulties in relationships. Furthermore, frustration about not being able to overcome these difficulties may lead to aggressive and dysfunctional behavior that in turn may reinforce both being and feeling alone. Therefore, it can be assumed that patients with antisocial PD are at higher risk to feel lonely.

There are very few loneliness studies in antisocial PD. Özdel et al. [27] assessed core beliefs about oneself and found that *N* = 38 participants with antisocial PD believed that they were more lonely, unlovable, and rejected compared to healthy controls. Furthermore, Ma et al. [28] found a positive correlation between loneliness and antisocial behavior, assessed as delinquency or aggressive behavior in *N* = 627 adolescents.

## Borderline Personality

Core characteristics of borderline PD (BPD) are a pervasive pattern of unstable interpersonal relationships, unstable and intensive affects, marked impulsivity, and an unstable self-image. Patients fear being abandoned, yet relationships rupture easily. Together with a chronic feeling of emptiness, patients with BPD are highly susceptible to feeling intensely lonely.

Multiple studies underline the clinical relevance of loneliness in patients with BPD that feel lonelier compared to healthy controls (*N* = 26 and *N* = 40, respectively [29, 30]). Furthermore, Liebke et al. [30] found that increased loneliness was correlated with smaller social networks and lower social functioning in BPD (especially in the domains of social engagement, prosocial behavior, and social communication). However, the authors conclude that social network characteristics and social functioning are not sufficient to explain loneliness in BPD. For instance, Lazarus and Cheavens [31] add the observation that female BPD patients (*N* = 27) had a less positive and less supportive perception of their network partners compared to healthy controls. Moreover, BPD patients (*N* = 32) showed a tendency to perceive others as more negative and less trustworthy compared to patients with social anxiety disorder and healthy controls [32]. Nenov-Matt et al. [33] replicated the findings of increased loneliness in BPD patients (*N* = 36) compared to healthy controls and found that loneliness was additionally correlated with increased rejection sensitivity, emotional abuse, and emotional neglect. The authors suggested rejection sensitivity as a mediator between childhood maltreatment and loneliness in adulthood.

Regarding loneliness and psychopathological symptoms, Mou et al. [34] found that the association between loneliness and suicidal ideation was specifically strong in psychiatric inpatients in case of the presence of a BPD diagnosis (*N* = 35). Furthermore, Slotema et al. [35] observed that loneliness showed a positive correlation with the severity of hallucinations in female borderline patients (*N* = 60). Buelens et al. [36] found that loneliness may represent a link between non-suicidal self-injury (NSSI) disorder and BPD in a sample of adolescents (*N* = 347) who had engaged in NSSI.

Finally, Schermer et al. [37] found that self-reported borderline personality features and loneliness were correlated in a large cohort of adult twins (*N* = 11,329). Whereas borderline personality features had a heritability estimate of *h*^2^ = 41% in this study, individual differences in loneliness had a heritability estimate of *h*^2^ = 36%. Thus, non-shared environmental factors contributing to this variance will need to be identified.

## Histrionic Personality

Patients with histrionic PD show a pervasive pattern of excessive emotionality and the need for attention from others. Therefore, being alone and not perceiving attention could represent a quite aversive state for histrionic PD. Furthermore, the rather loose social relationships may not sufficiently fulfill social needs. However, histrionic patients tend to overestimate intimacy and closeness of social relationships. As a result, it can be assumed that feelings of loneliness could be particularly strong in histrionic PD, yet the perceived supposedly closeness could buffer the perception of being alone and lonely.

Data on loneliness in histrionic PD is missing. Regarding dimensional measures, Berryman et al. [38] observed a negative association between loneliness and histrionic personality traits in young adults (*N* = 468) who use social media supporting the above-mentioned hypothesis.

## Narcissistic Personality

Narcissistic PD consists of a pervasive pattern of grandiosity (in fantasy or behavior), excessive need for admiration, and a lack of empathy resulting in difficulties in social relationships. Patients are preoccupied with their specialty and uniqueness and only feel understood by other high-status people. Loneliness may be common in narcissistic PD as patients do not feel understood and miss meaningful relationships with evenly matched others. However, patients with narcissistic PD may struggle to admit feeling lonely as this may represent a weakness.

Empirical loneliness studies in narcissistic PD are lacking, yet dimensional approaches underline a significant association of narcissism and loneliness. These dimensional approaches conceptualize narcissism as distinct factors, such as grandiosity and vulnerability or agentic extraversion, antagonism, and narcissistic neuroticism [39]. Whereas Sedikides et al. [40] report a negative relation between narcissism and loneliness if self-esteem is high in a series of studies, recent studies report mainly positive associations between narcissistic factors and loneliness. Kealy et al. [41] found that loneliness was positively associated with narcissistic vulnerability and to a lesser extent with grandiosity in young adults (*N* = 120). Furthermore, loneliness mediated between narcissistic vulnerability and life satisfaction. Similarly, Brailovskaia et al. [42] observed positive correlations between loneliness and grandiose and vulnerable narcissism (*N* = 701 participants). Both narcissistic factors moderated the association between loneliness and depressive symptoms. Rogoza et al. [43] further differentiated grandiosity in admiration and rivalry (antagonism) and found that vulnerable narcissism and rivalry positively predicted loneliness in *N* = 314 participants. In contrast, admiration negatively predicted loneliness. In line with this, Gąsiorowska et al. [44] reported that antagonistic narcissism was associated with increased loneliness and less social support in a community sample (*N* = 662). Finally, Carter and Douglass [45] observed that loneliness increased with age, whereas narcissism decreased in middle-aged and older-aged adults (*N* = 100 in each group). Furthermore, narcissism moderated the relationship between loneliness and age, leading to the authors’ conclusion that a certain degree of narcissism may even be protective against feeling lonely which underlines the relevance to further investigate the interplay of narcissism and loneliness.

## Avoidant Personality

Avoidant PD is characterized by a pervasive pattern of avoidance and inhibition in social situations due to feelings of inadequacy, hypersensitivity to criticism, and fear of rejection. Consequently, patients with avoidant PD can be assumed to be at a high risk to feel lonely as they easily withdraw from social interaction and tend to show restraint even within close relationships. Although patients may long for supportive relationships, this need for social support may be seen as a further personal inadequacy. Preoccupation with being rejected may additionally hinder desired close relationships and contribute to increased levels of loneliness in avoidant PD.

Available loneliness data for avoidant PD is limited. Using avoidant PD diagnostic criteria as a dimensional score, Hayakawa et al. [46] found that avoidant personality features were positively associated with loneliness, social anxiety, and mistrust of others, and negatively associated with social support in non-clinical participants (*N* = 101). Yuan et al. [47] reported that loneliness was correlated with social avoidance tendencies in college students (*N* = 1021). Furthermore, loneliness and interpersonal trust chain-mediated between social avoidance and depression.

Notably, avoidant PD shows an important overlap with social anxiety disorder [48]. Several studies found that social isolation and loneliness are common in social anxiety disorder (for a review and meta-analysis, see [49]). In addition, it was shown that a reduction in social anxiety in patients with social anxiety disorder (*N* = 108) was associated with decreased loneliness after treatment [50]. In contrast, treatment studies for avoidant PD are limited [48] and lack data regarding effects on loneliness.

## Dependent Personality

Patients with dependent PD are unable to be alone and rely on others for reassurance and support. They urge to seek another relationship when one ends and fear losing social support. As a consequence, it can be assumed that patients fear the feeling of loneliness, are at high risk to feel lonely when being alone, and experience loneliness as a highly aversive state [51].

Research on loneliness in dependent PD is limited to dimensional assessments. For instance, Overholser [52] found that loneliness was positively correlated with dependent personality features in psychiatric inpatients (*N* = 43) and nonpatient adults (*N* = 66). Pritchard and Yalch [53] found a positive association between dependency and loneliness in college students (*N* = 176). Similarly, Besser et al. [54] reported a positive association between dependency and loneliness in college students (*N* = 462) during the COVID-19 pandemic.

## Obsessive–Compulsive Personality

Obsessive–compulsive PD is characterized by a pervasive pattern of preoccupation with orderliness and perfectionism. Patients have an excessive need for control, seem inflexible, and show extreme devotion to work. Consequently, it can be assumed that patients are at a higher risk of feeling lonely as they fear being rejected for not being perfect. Furthermore, patients may fail in installing close and warm relationships as rigidity may reject others and relationships cannot be controlled as desired.

There is a lack of studies that assess loneliness in obsessive–compulsive PD or traits. Solomonov et al. [55] assessed the interpersonal profiles of *N* = 43 patients with obsessive–compulsive PD and identified two distinct clusters (domineering-vindictive or submissive-exploitable). Especially the first pattern can be assumed to challenge relationships, elicit rejection, and result in isolation and loneliness.

Interestingly, the related dimensional Big Five personality trait of conscientiousness was negatively associated with loneliness in a recent meta-analysis [56•]. It could be assumed that individuals high in this trait may conscientiously maintain their social relationships. Although obsessive–compulsive PD and specific facets of conscientiousness may overlap, this finding may not be generalizable as obsessive–compulsive PD may not represent the extreme variant of conscientiousness [57].

## Loneliness and Alternative Model of Personality Disorder

Novel dimensional approaches like AMPD further enable clarification of associations between loneliness and specific personality traits. So far, two studies regarding criterion B (trait domains) were identified. Romero and Alonso [58•] found that loneliness was significantly positively associated with negative affectivity (i.e., emotional lability, separation insecurity, anxiousness), detachment (i.e., withdrawal, intimacy avoidance, anhedonia), and psychoticism (i.e., eccentricity, unusual cognitions) in adolescents (*N* = 921) before and after controlling for age and sex. No association with antagonism or disinhibition was observed. Similarly, Roche et al. [59•] found that loneliness measured daily (“Today I felt lonely”) correlated significantly with negative affectivity and detachment but not psychoticism, antagonism, or disinhibition in adult college students (*N* = 248).

Studies assessing the association of loneliness with criterion A (impairment in self and interpersonal functioning) are still lacking. However, it can be assumed that impairment in domains like identity, self-direction, empathy, and intimacy may be associated with loneliness.

## Conclusion

Our synopsis of empirical loneliness studies in PD found that the number of studies varied greatly for different PDs, i.e., the majority of studies was available for BPD underlining its observed clinical relevance in this condition. For several PDs, studies on loneliness are only available for the respective (subclinical) personality traits, but not for full diagnostic PD entities. In general, loneliness seems to increase in PDs; however, also exceptions were found. For instance, our findings suggest that histrionic personality is associated with a reduction of loneliness. In addition, the intensity of loneliness seems to vary with the severity of psychopathological symptoms such as negative affect, depressive symptoms, social withdrawal, paranoid beliefs, hallucinations, and suicidality. As a consequence, loneliness may further reduce life satisfaction in PD and contribute to the overall burden of disease. Studies are lacking that compare the intensity of loneliness in different PDs and/or use the dimensional approach of the AMDP criterion A. Finally, our findings underline a need for longitudinal studies that analyze the interplay of PD and loneliness over time.

An open question remains how loneliness and PD exactly interplay and whether loneliness is resulting from PD. As impairment of self and interpersonal functioning is associated with PD per definition [11], patients with PD often have difficulties in deeply or stably connecting with others, experience this deficit in daily life, and may feel less connected or even excluded and rejected [9••]. On an intrapersonal level, patients with PD may have difficulties in recognizing, mentalizing, and regulating their own emotions [11] and may have a reduced capacity to cope with loneliness, ultimately resulting in more intense and longer-lasting aversive states of loneliness. Furthermore, various PDs may differ regarding their experience of belonging and PD-specific factors may enforce the perception of loneliness by different mechanisms (e.g., fear of rejection in avoidant PD vs. feeling not understood and seen in narcissistic PD). Additionally, higher rates of perceived discrimination and internalized stigma of PD may contribute to loneliness in PD [10]. On an interpersonal level, dysfunctional emotional and behavioral patterns associated with PD (e.g., impulsivity, mistrust, and hostility in case of BPD, paranoid or narcissistic PD) may elicit rejection by others and challenge relationships. A limited inflexible repertoire of social skills to repair ruptured relationships (e.g., BPD) and the inability to admit one’s own mistakes (e.g., narcissistic PD) may further hamper maintaining stable and close relationships. Consequently, the social network size may decrease, the quality of social relationships may suffer, and social support may be lost. Being alone and isolated may further increase the risk of feeling lonely as weak to moderate correlations between social isolation and loneliness were observed [60, 61]. Finally, the behavioral impulse of social withdrawal associated with several PDs may contribute to social isolation and loneliness.

**Fig. 1:**
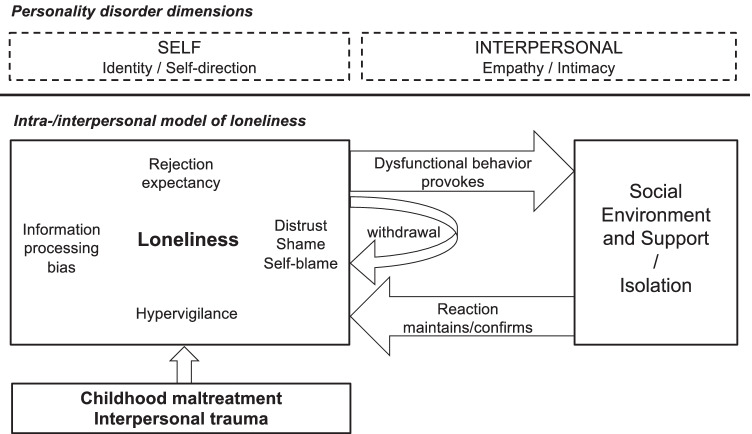
Theoretical model of loneliness in PD: Impairment in dimensions of self and interpersonal functioning according to the DSM-5 Alternative Model of Personality Disorders parallels intra- and interpersonal factors of loneliness. Childhood maltreatment and interpersonal trauma may predispose to feel lonely. Interpersonally, social withdrawal and dysfunctional behavior may provoke social network impairment, rejection, and loss of social support that maintains loneliness. Intrapersonal factors such as rejection sensitivity (hypervigilance to social cues and rejection expectancy), information processing biases, distrust, shame (e.g., internalized stigma), and self-blame further hinder reconnection attempts to overcome loneliness. Specific dysfunctional personality traits (e.g., detachment and negative affectivity) need to be identified that further interplay with these intra- and interpersonal factors and increase loneliness. Intra-/interpersonal model of loneliness adapted from [33, 81]

Loneliness research has identified intra- and interpersonal factors that contribute to persisting loneliness and overlap with the above-mentioned PD facets. Due to shared intra- and interpersonal factors (see Fig. 1 for a comprehensive model), loneliness and PD may collude in a “complex vicious cycle, in which loneliness and difficulties in managing interpersonal relationships are mutually reinforcing” (page 13, [9••]). For instance, loneliness is associated with hypervigilance to social cues of rejection [2, 33, 62]. This rejection sensitivity further encompasses the anxious expectation of being rejected, the ready perception, and the overreaction to rejection [63]. High rejection sensitivity is found in BPD [64], narcissistic PD [65], and avoidant PD (preoccupation with rejection as one criterion), and may constitute an important link between loneliness and PDs. Furthermore, loneliness has been associated with several information processing biases such as a negative self- and other-evaluation, a self-defeating attribution style, and reduced social self-efficacy (for an overview, see [66]). Altered social cognition has been observed in PD as well which may at least partly overlap with findings in loneliness. For instance, BPD patients seem to rate others less trustworthy [32], negatively process social cues that signal affiliation compared to healthy controls [67], and feel more readily excluded [68, 69]. In addition, these biases may concern the perception of social support, e.g., beliefs about potentially available support. Interestingly, reduced perceived social support has been associated both with loneliness [70] and with PDs [71]. Loneliness may lead to initial withdrawal in order to prepare for social reconnection attempts [2]. However, withdrawal may be maintained by intrapersonal processes such as increased rejection sensitivity and feelings of distrust, self-blame, and shame (e.g., internalized stigma) that are similarly observed in PD and hinder reconnection. Finally, loneliness has been associated with a more hostile-submissive behavior [72] that may—similarly to PD—elicit anticipated rejection. Taken together, loneliness may contribute to PD severity due to shared intra- and interpersonal factors.

Loneliness and PD may further share common roots in childhood maltreatment [9••, 33] characterized by emotional abuse, emotional neglect, and frustrated emotional needs of intimacy and attachment that may predispose to feeling lonely later in life (see Fig. 1). Childhood maltreatment may lead to the development of rejection schemes, negative cognitions about belonging, and insecure attachment styles that are associated both with PDs [73] and with loneliness [74]. Yet, longitudinal assessments are necessary to analyze this complex interplay that should additionally include the assessment of psychopathological symptoms. For instance, loneliness is highly intertwined with depressive symptoms [75, 76] and reported by patients with depressive [76] or psychotic disorder [77]. Therefore, the experience of loneliness is not limited to PD, and a more thorough investigation of loneliness as a transdiagnostic construct is needed.

Finally, both PD and loneliness show a quite stable and pervasive pattern with limited treatment options. Whereas disorder-specific psychotherapy was found to be effective in treating specific PD symptoms (for instance, BPD symptom severity [78]), loneliness is not a standard outcome in most treatment studies and is easily overseen in clinical routine. Treatment approaches such as schema therapy for PD that addresses aversive feelings such as loneliness stemming from unmet childhood needs [79, 80], Cognitive Behavioral Analysis System of Psychotherapy [81], or the use of group therapy [9••] may be promising. Randomized-controlled trials and dismantling designs are necessary to identify effective loneliness interventions that can be incorporated into psychotherapeutic treatment.

Taken together, loneliness as a unitary construct is common in PD and may contribute to its severity and persistence. Several shared intra- and interpersonal factors may explain the association between loneliness and specific PDs that need to be clarified to effectively address loneliness in PD. Dimensional PD approaches such as AMPD could further help to identify underlying mechanisms across different PDs (e.g., the role of detachment). Future research needs to focus on common origins of loneliness and PD in childhood maltreatment, identify maintaining factors (e.g., rejection sensitivity), differentiate the facets of loneliness and related factors and symptoms (e.g., inner emptiness), and use longitudinal and experimental designs (especially regarding treatment options).
